# Reliability of Addenbrooke's Cognitive Examination III in differentiating between dementia, mild cognitive impairment and older adults who have not reported cognitive problems

**DOI:** 10.1007/s10433-021-00652-4

**Published:** 2021-09-22

**Authors:** C. Potts, J. Richardson, R. B. Bond, R. K. Price, M. D. Mulvenna, P. Zvolsky, M. Harvey, C. F. Hughes, F. Duffy

**Affiliations:** 1 Faculty of Computing, School of Computing, Engineering and the Built Environment, Ulster University, Newtownabbey, Coleraine, UK; 2 Memory Service, Northern Health and Social Care Trust, Antrim, UK; 3 Faculty of Life and Health Sciences, School of Biomedical Sciences, Ulster University, Coleraine, UK

**Keywords:** Cognitive screening test, Cognitive assessment, Alzheimer’s disease, Vascular dementia, MCI, NHSCT Memory Service

## Abstract

Diagnosing dementia can be challenging for clinicians, given the array of factors that contribute to changes in cognitive function. The Addenbrooke’s Cognitive Examination III (ACE-III) is commonly used in dementia assessments, covering the domains of attention, memory, fluency, visuospatial and language. This study aims to (1) assess the reliability of ACE-III to differentiate between dementia, mild cognitive impairment (MCI) and controls and (2) establish whether the ACE-III is useful for diagnosing dementia subtypes. Client records from the Northern Health and Social Care Trust (NHSCT) Memory Service (*n* = 2,331, 2013–2019) were used in the analysis including people diagnosed with Alzheimer’s disease (*n* = 637), vascular dementia (*n* = 252), mixed dementia (*n* = 490), MCI (*n* = 920) and controls (*n* = 32). There were significant differences in total ACE-III and subdomain scores between people with dementia, MCI and controls (*p* < 0.05 for all), with little overlap between distribution of total ACE-III scores (< 39%) between groups. The distribution of total ACE-III and subdomain scores across all dementias were similar. There were significant differences in scores for attention, memory and fluency between Alzheimer’s disease and mixed dementia, and for visuospatial and language between Alzheimer’s disease–vascular dementia (*p* < 0.05 for all). However, despite the significant differences across these subdomains, there was a high degree of overlap between these scores (> 73%) and thus the differences are not clinically relevant. The results suggest that ACE-III is a useful tool for discriminating between dementia, MCI and controls, but it is not reliable for discriminating between dementia subtypes. Nonetheless, the ACE-III is still a reliable tool for clinicians that can assist in making a dementia diagnosis in combination with other factors at assessment.

## Introduction

As people get older, they experience changes in cognitive function some of which are associated with normal ageing. One of the challenges in clinical practice is to differentiate between presentations that are consistent with functional cognitive impairment, mild cognitive impairment (MCI), a dementia or something else. In most cases, those with cognitive impairments will be referred to specialist memory services. Specialist memory services offer timely differential diagnosis, which is beneficial for the person as it allows for better adjustment, slowing of progression and planning ahead, and there are also significant savings to the health economy (Bamford et al. 2004; Prince et al. 2011; Pratt and Wilkinson 2003; Banarjee and Wittenberg 2009).

There is no single test for dementia, and diagnosis is made on the basis of excluding other causes for the symptoms and clinical impression. Dementia assessment at a specialist service generally involves a formal assessment of cognitive function, activities of daily living, social, educational and employment history and a collateral history from someone who knows the person well. The person may also be referred for brain imaging. All of this information is reviewed to help make a differential diagnosis. It is most likely that clinicians, through clinical experience, weight the relative contribution of each of the multiple sources of information to come to a decision about diagnosis; however, there is no agreed weighting for this information. There is also no agreed process across services which means that there is significant variability in the assessments used at different services. Taken together, this leaves the potential for diagnostic variability across services.

Cognitive profiles vary across MCI and the different types of dementia. Those living with MCI typically experience cognitive impairment between that of normal ageing and mild dementia (Grundman et al. 2004). Amnestic MCI typically presents with predominant impairment in memory with increased likelihood of progression to Alzheimer’s (Grundman et al. 2004) while in non-amnestic MCI memory is preserved but one other cognitive domain will be affected. People with Alzheimer’s will have impairment in memory and at least one other cognitive domain such as attention, language, visuospatial ability and fluency. Greater impairment with episodic memory is seen in people with Alzheimer’s compared to those with vascular dementia (Graham and Hodges 2004; Karantzoulis et al. 2011). In contrast, people with vascular dementia have worsening semantic memory, attention and visuospatial functioning in comparison to people with Alzheimer’s (Graham and Hodges 2004).

Cognitive assessment is a key factor in decision making, and part of this process involves screening. The use of screening tools alone will not determine the diagnosis; however, the choice of test is still important. Different screening tools are used across services such as the Montreal Cognitive Assessment (MoCA) (Nasreddine et al. 2005), Mini-Mental State Examination (MMSE) (Folstein et al. 1975) and the Addenbrooke’s Cognitive Examination III (ACE-III). The original ACE was developed to help detect mild dementia and differentiate between Alzheimer’s disease and frontotemporal dementia (Mathuranath et al. 2000). It was initially designed as an extension to the commonly used MMSE, with additional neuropsychological domains incorporated to improve screening performance (Mathuranath et al. 2000), and was later revised (ACE-R) with clearly defined subdomain scores (Hodges and Larner 2016). The ACE-III was subsequently created to remove elements of the MMSE and address weaknesses of the ACE-R (Hsieh et al. 2013). The ACE-III takes around half an hour to complete and is scored out of 100, with higher scores corresponding to better cognitive function. It incorporates five subdomains: attention, memory, fluency, language and visuospatial. The ACE-III has been validated as a screening tool for cognitive deficits in Alzheimer’s disease and frontotemporal dementia and has been translated and validated in other languages including Chinese, Japanese and Spanish (Wang et al. 2017; Li et al. 2019; Takenoshita et al. 2019; Matias-Guiu et al. 2015). The ACE-III, MoCA and MMSE have all been recommended by the Department of Health and the Alzheimer’s Society in the UK for inclusion as part of a comprehensive cognitive assessment in memory clinics (Ballard et al. 2015). However, the ACE-III can more accurately detect frontotemporal dementia, as well as the earlier stages of dementia compared to MMSE (Hsieh et al. 2013; Slachevsky et al. 2004). In addition, ACE-III is better at identifying everyday activity impairments in dementia when compared to MMSE and MoCA (Giebel and Challis 2017). Thus, the results from these studies would suggest that ACE-III is preferrable when compared to other screening tools. While the ACE-III is commonly used as part of a full clinical assessment, few studies have looked at how reliable the ACE-III is alone for distinguishing between dementia and MCI, and the different types of dementia.

The aim of this study is to assess how reliable the ACE-III assessment is for making a differential diagnosis between dementia, MCI and controls. Therefore, this study seeks to address the following questions: can the ACE-III help to differentiate between dementia, MCI and older adults who have not reported cognitive problems? For a person with dementia, is the ACE-III helpful in differentiating the type of dementia?

## Methods

### Memory service

The Northern Health and Social Care Trust (NHSCT) in Northern Ireland set up a memory service in 2013 to facilitate timely diagnosis and enable people with dementia to access appropriate supports. The NHSCT Memory Service accepts referrals for people who present with symptoms of memory problems and/or behavioural change within a clinical picture suggestive of a dementia. Since 2013, there have been over 6000 referrals to the NHSCT Memory Service, however not everyone who attends for assessment has dementia. Potential outcomes of assessment include (1) diagnosis of a specific type of dementia, (2) diagnosis of MCI, (3) another condition which causes changes in cognitive function treatable or untreatable and (4) no evidence of a physical or mental health condition. The comprehensive assessment process in the NHSCT Memory Services involves a review of the following factors:

- Background information (education level, occupational history, family history of dementia, establishing whether the person can recall their own personal history)
- Symptoms (do they have insight into their own symptoms, did the person self-report cognitive difficulties, did their carer report cognitive difficulties, onset of symptoms, progression of symptoms, known previous psychiatric history, current mental health problems/ stressors, sleep problems, history of self-harm/ suicidal ideation, hallucinations, delusions, psychosis)
- General information (impairment in activities of daily living, can they drive, are they financially independent or do they require support, living situation)
- Health information (do they require assistance with medication, any history of falls, mobility/ movement problems, difficulty hearing, problems with eyesight, if they smoke or drink alcohol)
- Physical health risk factors (epilepsy, head injury, heart disease, stroke/ cerebrovascular accident/ transient ischaemic attacks, high blood pressure, recurrent infections, diabetes, peptic ulcer, high cholesterol, Asthma/ COPD, neurological)
- Tests (ACE-III, Bristol Activities of Daily Living, Zarit Caregiver Burden)

A formal diagnosis can then be made by the psychiatrist based on the assessment of the aforementioned factors. Psychiatrists in the NHSCT Memory Service use ICD-10 criteria to classify dementia type. However, the ICD-10 codes are not recorded in the database.

### Data provenance

**Fig. 1:**
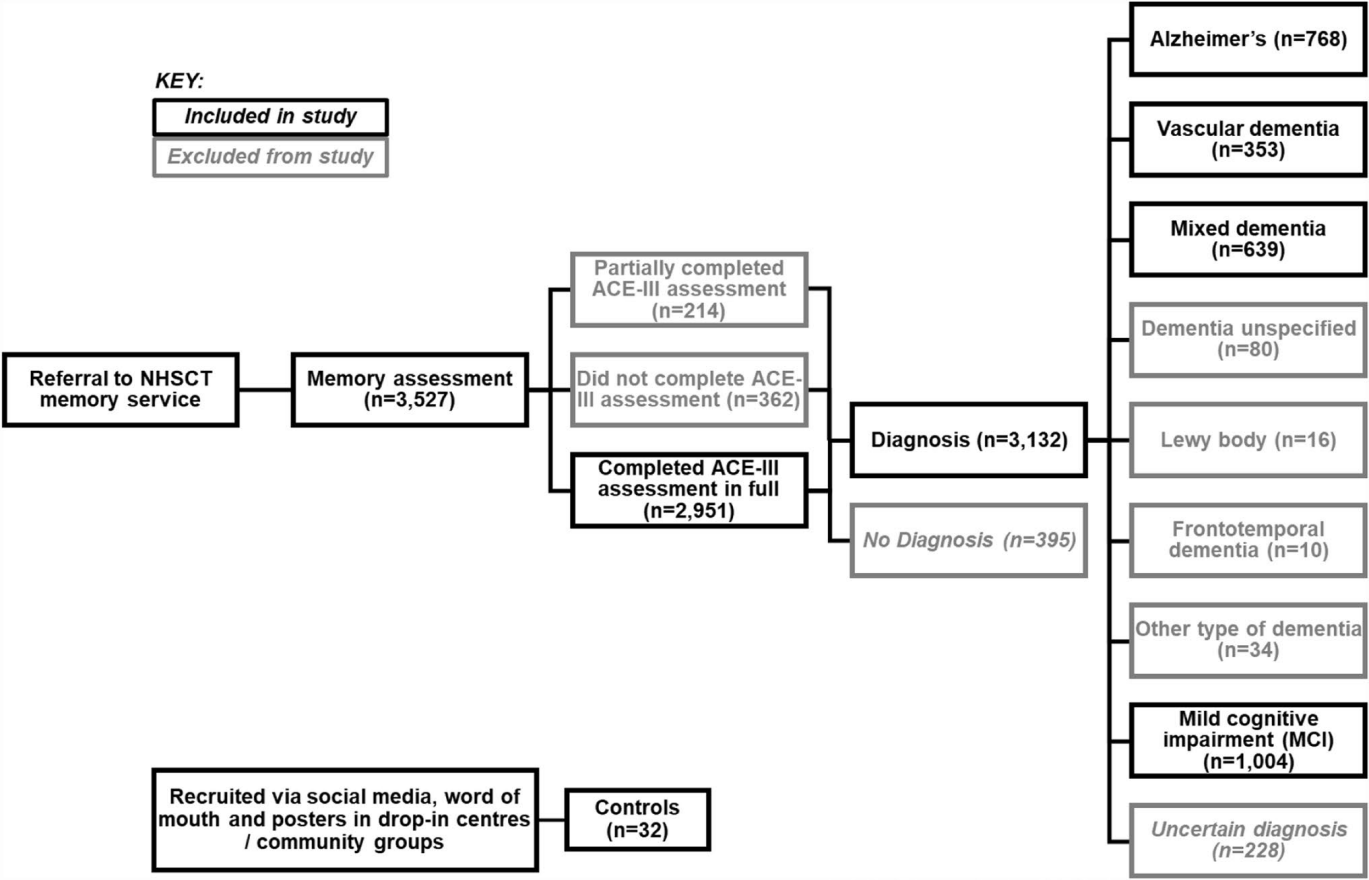
Overview of the person’s journey with the NHSCT Memory Service, including those who were included and excluded from the study. Additional path shown for control participants who were not referred to the memory service but were recruited to the study (bottom left)

This study received ethical approval from the Health Research Authority ethics board (ref: 17/NI/0142). Data were obtained from the NHSCT Memory Service. Over 3500 patient records from dementia assessments were digitised from 2013 to 2019. An overview of the person’s journey through the service is shown in Fig. 1. Most people were referred to the memory service by their GP (93%), while others were referred by other medical professionals or mental health services (7%). Once referred, people attend for a comprehensive dementia assessment where they may receive a diagnosis of dementia, mild cognitive impairment (MCI), other diagnosis or no diagnosis (Fig. 1).

The outcomes of memory assessment are as follows.

- *No diagnosis* This group received no diagnosis of dementia or MCI. These individuals may have one of a range of presentations, for example: mental health problems, CVA/ brain injury, other cognitive impairment, age-related changes in cognitive function, Parkinson’s disease, other conditions or no evidence of physical or mental health difficulty.
- Those with a specific diagnosis recorded, including *Alzheimer’s disease, vascular dementia, mixed dementia, Lewy body dementia, frontotemporal dementia* or *MCI*
- *Dementia unspecified* These people have presentations consistent with dementia, but at the time of assessment the exact type of dementia was unclear. These people are typically reviewed by a clinician at a later date and given a diagnosis.
- *Other type of dementia* Those diagnosed with another type of dementia, not listed above. The exact type of dementia was not recorded at time of assessment.
- *Uncertain diagnosis* This group did not receive a definitive diagnosis at the time of assessment. In the database, this was recorded as between two or more diagnoses. These people are typically reviewed by a clinician at a later date and given a diagnosis.

### Participants and exclusion criteria

Data were filtered to only those that completed the ACE-III in full; therefore, those who partially completed (*n* = 214) or did not complete the assessment (*n* = 362) were excluded from the present study. All individuals in the ‘no diagnosis’ of MCI or dementia category (*n* = 351) were excluded given the range of presentations in this cohort as mentioned above. Those that were diagnosed with Lewy body dementia (*n* = 15) and frontotemporal dementia (*n* = 8) were removed due to small sample sizes. Those in the dementia unspecified category (*n* = 59), other types of dementia (*n* = 24) and those who received an uncertain diagnosis (*n* = 228) were omitted as the exact diagnosis was not recorded at the time of assessment.

Additionally, a group of older adults (*n* = 32; ≥ 65 years) who had not presented to the memory service and did not have a diagnosis of dementia were recruited via social media, word of mouth and posters in drop-in centres or community groups, to provide comparative ‘control’ data. These participants completed the same dementia assessment, including the ACE-III, administered by a trained memory service practitioner using similar protocols to those employed in the memory service. The intention was to recruit 100 individuals with no reported cognitive problems as controls for the study; however, this sample size was not achieved due to the COVID-19 pandemic.

The final study cohort included 2,331 data records on 2,176 people. A total of 2,023 (93%) people used the service only once, of which 151 (6.9%) used the service twice and 2 (0.1%) people attended three times.

### Data analysis

R programming language and RStudio (version 3.6.0) were used for all data analyses. Exploratory analysis was carried out to investigate age, sex, total ACE-III score and scores for the ACE-III domains (attention, memory, fluency, language and visuospatial). ACE-III total and domain scores for the diagnostic groups were visualised using boxplots and density plots and were assessed for normality using Shapiro–Wilk tests. For all features, *p* < 0.05 which suggested the data was not normally distributed. This was confirmed by visual inspection of histograms/ boxplots, indicating nonparametric testing should be applied. Kruskal–Wallis tests were performed across diagnostic groups for age, ACE-III total and domain scores, with *p* < 0.05 considered to be statistically significant. Post hoc pairwise Wilcoxon rank sum tests were carried out using Bonferroni correction for multiple testing. A Chi-squared test was used to compare proportions of gender across diagnostic groups. Violin plots were produced, which combine the boxplot and density plot to better illustrate summary statistics and distribution in one plot (Hintze and Nelson 1998). Pairwise comparisons for total ACE-III score and ACE-III domain scores across diagnostic groups were visualised using a tile plot, with statistical significance shown for each comparison.

Plots showing estimated kernel densities were produced to compare total ACE-III score for all dementias (Alzheimer’s disease, vascular dementia and mixed dementia), MCI and controls. Based on the results of the pairwise comparisons that were significant, additional density plots were produced for a subset of the ACE-III domains across dementia diagnoses. The overlapping coefficient, which is the overlapping area under two probability density functions, was calculated for each of these comparisons.

As the maximum score differs for each ACE-III domain, scores were normalised by rescaling the data points between 0 and 100 (Eq. 1).

1 $$x_{{{\text{norm}}}} = \frac{{x_{{\text{i}}} - {\text{min}}\left( x \right)}}{\max \left( x \right) - \min \left( x \right)}*100$$

Eq. 1: Formula for normalisation where x_i_ is a data point (x_1_, x_2_…x_n_) and x_norm_ is a normalised data point.

For each domain, the normalised mean scores were visualised using a line plot for comparisons across diagnostic groups. The relative differences between mean scores across each of the ACE-III domains were compared for all dementias, MCI and controls. Kruskal–Wallis tests were performed to compare ACE-III domain scores for all dementias, MCI and controls, with *p* < 0.05 considered to be statistically significant. Post hoc pairwise Wilcoxon Rank Sum tests were carried out using Bonferroni correction for multiple testing.

Receiver operating characteristic (ROC) curves were plotted to assess the optimal cut-off points for the detection of MCI and dementia from controls, and dementia from MCI. Maximum Youden index (Youden index = sensitivity + specificity–1) was used to determine the optimal cut-offs.

## Results

Over half of people in this study were female (62%), with a smaller proportion of males (38%), and the average age was 79.6 (SD 7.5). Age was significantly different across diagnostic groups (*p* < 0.001, Table 1). Post hoc testing revealed that there were no significant differences in mean age across the dementia groups; however, people diagnosed with MCI were significantly younger on average compared to the dementia groups (*p* < 0.05), and the control group were also significantly younger on average (*p* < 0.05) compared to the MCI and dementia groups. Across diagnoses, proportions of gender were significantly different (*p* < 0.001, Table 1). Over 70% of people diagnosed with Alzheimer’s disease were female and around 60% of people with mixed dementia, MCI and controls were female (Table 1). Vascular dementia was the only group with almost even split of male and female (Table 1).

**Table 1:** Memory service client demographic information and ACE-III scores (*N* = 2,331)

		Alzheimer’s disease (*n* = 637)	Vascular dementia (*n* = 252)	Mixed dementia (*n* = 490)	MCI (*n* = 920)	Control *(*n* = 32)	Chi- square (df)	P-value
Age	Range Mean (SD)	47–100 80.4 (7.5)	56–98 81.4 (7.1)	59–102 81.6 (6.3)	51–100 77.5 (7.7)	65–91 77.0 (7.2)	139.6 (4)	< 0.001^a^
Sex, N (%)	Male Female	188 (29.5) 449 (70.5)	120 (47.6 132 (52.4)	186 (38.0) 304 (62.0)	371 (40.3) 549 (59.7)	13 (40.6) 19 (59.4)	31.6 (4)	< 0.001^b^
Education level, N (%)	Up to age 14/ no qualifications Up to A-level/ tech Degree Higher degree Unknown	381 (59.8) 141 (22.1) 45 (7.1) 5 (0.8) 65 (10.2)	161 (63.9) 49 (19.4) 9 (3.6) 2 (0.8) 31 (12.3)	326 (66.5) 92 (18.8) 24 (4.9) 2 (0.4) 46 (9.4)	510 (55.4) 242 (26.3) 58 (6.3) 10 (1.1) 100 (10.9)	10 (31.2) 9 (28.1) 7 (21.9) 6 (18.8) 0 (0)	140.5 (16)	< 0.001^b^
ACE-III total (max. 100)	Range Mean (SD)	16–90 56.6 (13.8)	17–90 53.8 (15.3)	7–84 54.0 (13.8)	24–97 70.2 (11.6)	64–98 85.7 (9.2)	642.1 (4)	< 0.001^a^
ACE attention (max. 18)	Range Mean (SD)	3–18 11.6 (3.6)	2–18 11.4 (3.6)	0–18 11.0 (3.4)	5–18 14.7 (2.7)	10–18 16.7 (1.8)	534.6 (4)	< 0.001^a^
ACE memory (max. 26)	Range Mean (SD)	0–25 9.5 (4.4)	0–23 9.9 (4.9)	0–20 9.1 (4.3)	0–26 13.9 (4.8)	12–26 20.5 (4.7)	500.4 (4)	< 0.001^a^
ACE fluency (max. 14)	Range Mean (SD)	0–12 5.0 (2.7)	0–12 4.4 (2.7)	0–12 4.5 (2.7)	0–14 6.7 (2.7)	5–13 9.5 (2.3)	321.7 (4)	< 0.001^a^
ACE language (max. 26)	Range Mean (SD)	2–26 18.8 (4.6)	1–26 17.6 (5.2)	0–26 18.3 (4.7)	3–26 21.6 (3.6)	18–26 24.4 (2.1)	350.5 (4)	< 0.001^a^
ACE visuospatial (max. 16)	Range Mean (SD)	0–16 11.6 (3.0)	0–16 10.5 (3.2)	0–16 11.2 (2.9)	0–16 13.3 (2.4)	12–16 14.6 (1.5)	316.5 (4)	< 0.001^a^

*older adults who have not reported cognitive problems

^a^Kruskal–Wallis p-value

^b^Chi-squared *p*-value

### Comparison of total ACE-III and domain scores across diagnoses

**Fig. 2:**
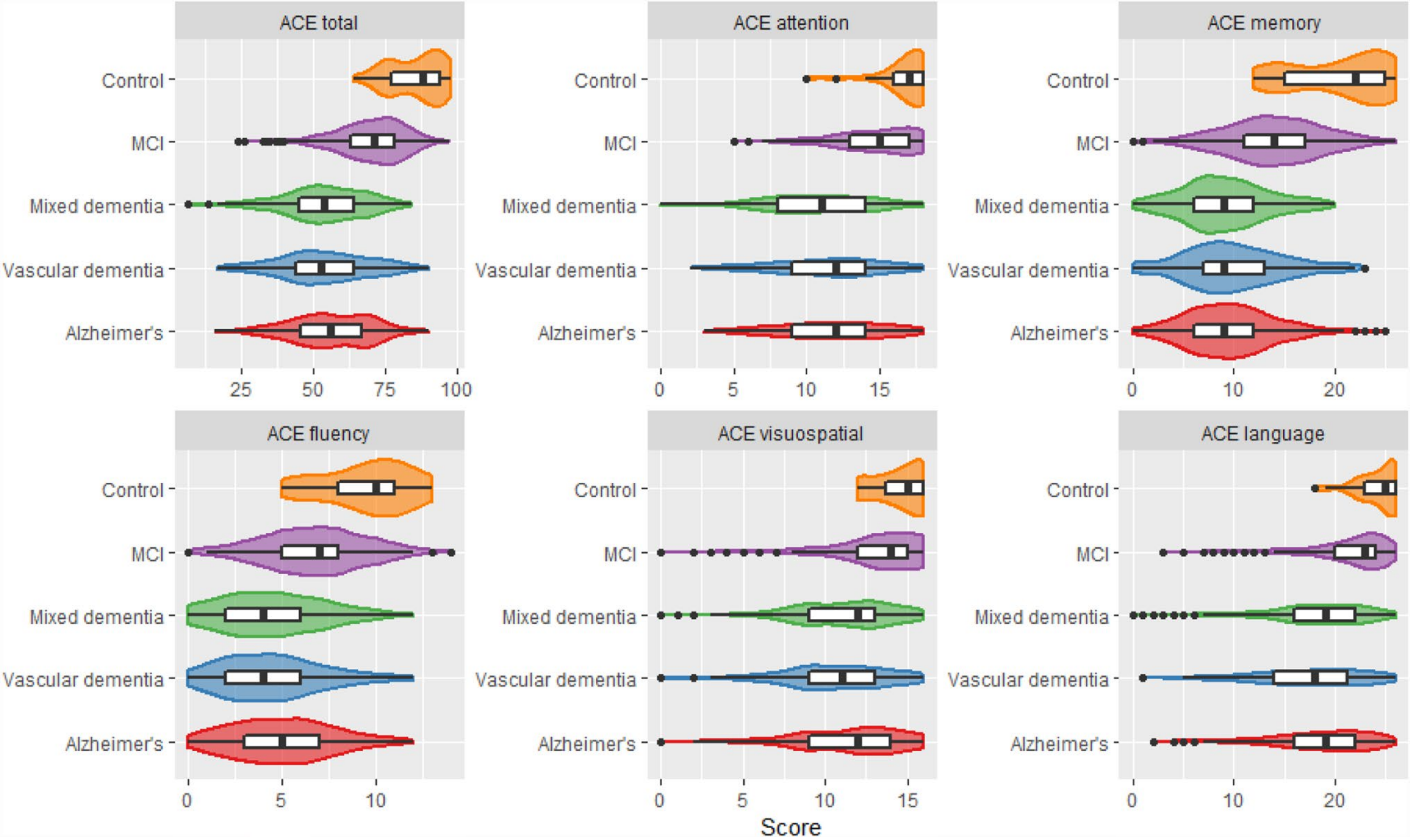
ACE-III total and domain scores across diagnoses presented as violin plots. Centre boxplots indicate median, quartiles, whiskers and outliers

ACE-III total and domain scores were significantly different across diagnoses, with higher average scores for the MCI and control groups compared to the dementia groups (Table 1). Overall, the scores for the control group were much higher than the MCI and dementia groups across all domains and total ACE-III (Fig. 2). The distribution of ACE-III total and domain scores was similar across all dementia groups (Fig. 2). People with Alzheimer’s disease displayed higher median scores for total ACE-III, fluency, visuospatial and language compared to those in the mixed and vascular dementia groups (Fig. 2). The MCI group attained scores in between those with dementia and the controls (Fig. 2). There was some overlap between the lowest recorded scores for those with MCI compared to the dementia groups; however, in general the scores were centred around the upper ranges of the dementia groups (Fig. 2). The distribution of scores for ACE-III total and memory for the controls was bimodal while for all other ACE-III domains the distribution was negatively skewed given most of the scores were high (Fig. 2).

**Fig. 3:**
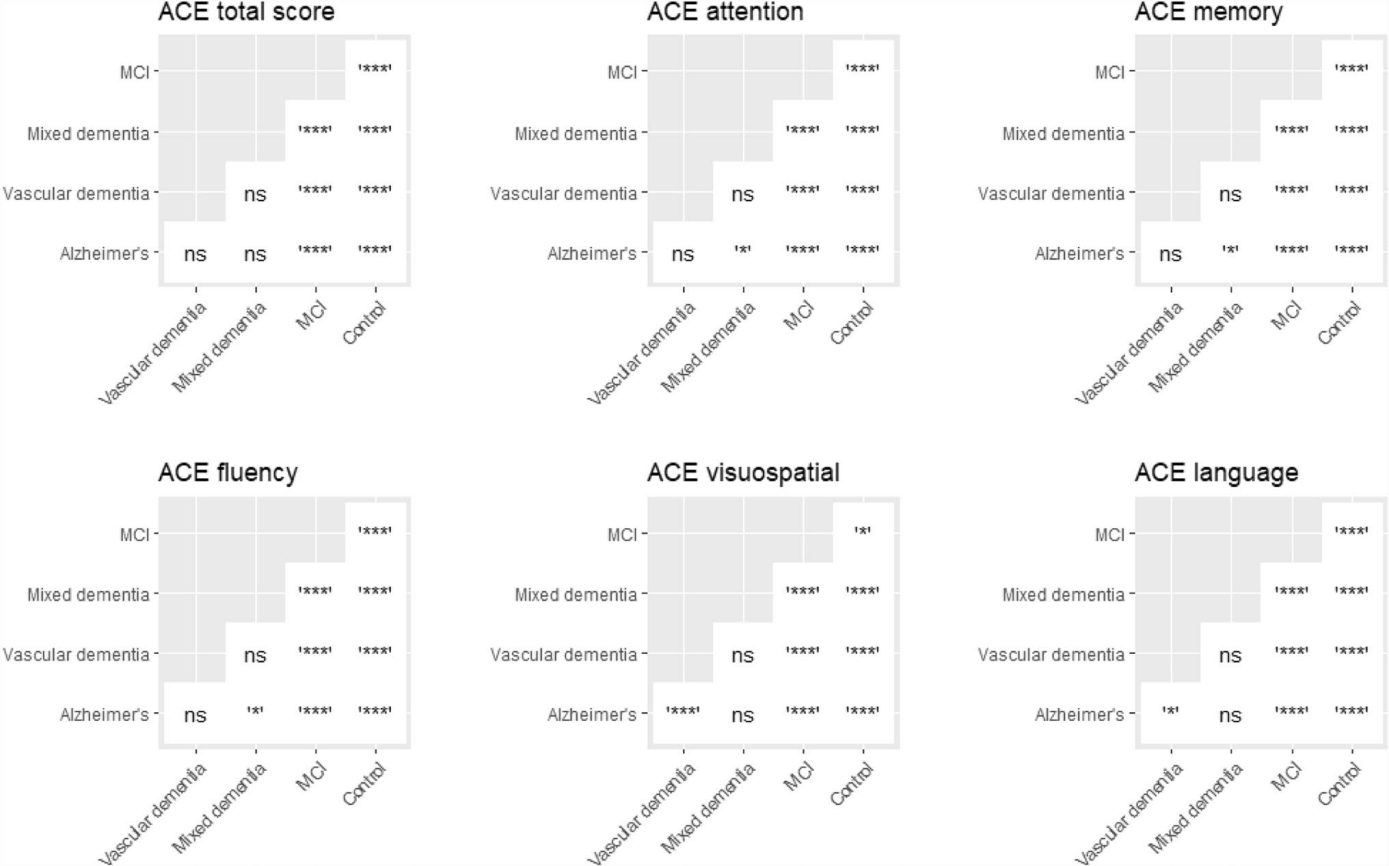
Pairwise comparisons for ACE-III total and domain scores across diagnoses. Significance codes; ns: *p* > 0.05, *: *p* <  = 0.05, **: *p* <  = 0.01, ***: *p* <  = 0.001, ****: *p* <  = 0.0001. All results adjusted for multiple testing using Bonferroni correction

Pairwise comparisons revealed significant differences in total ACE-III and domain scores between MCI and controls; all dementia groups and controls; MCI and all dementia groups; (Fig. 3, *p* < 0.05 for all). Significant differences in scores were present between mixed dementia and Alzheimer’s disease for attention, memory and fluency (Fig. 3). Scores for visuospatial and language were significantly different between vascular dementia and Alzheimer’s disease groups (Fig. 3).

### ACE-III cut-off analysis

**Fig. 4:**
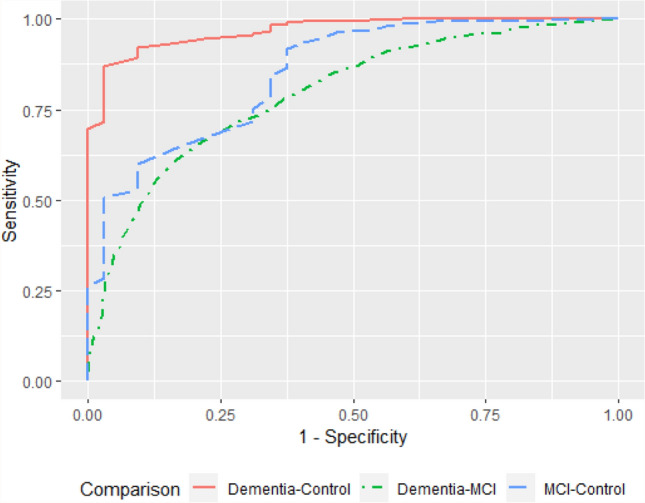
ROC curve of ACE-III for detecting dementia, MCI and controls

The optimal cut-off for differentiating dementia from controls based on the maximum Youden index is 71, with acceptable sensitivity (0.87) and high specificity (0.97) (Fig. 4, Table 2). When distinguishing dementia from individuals with no cognitive impairment, the recommended cut-off of 88 for screening purposes yielded high sensitivity (0.99) but very poor specificity (0.48). At the lower cut-off of 82 recommended for research, specificity improved slightly (0.63) with comparable sensitivity (0.97). The optimal cut-off for distinguishing MCI from controls was 84 with high sensitivity (0.92) but low specificity (0.63) (Fig. 4, Table 2). A cut-off of 61 was identified as optimal for differentiating dementia from MCI; however, the sensitivity and specificity were poor at 0.66 and 0.79, respectively (Fig. 4, Table 2).

**Table 2:** Optimal cut-off scores for ACE-III

Comparison	Optimal cut-off	AUC	Youden index	Sensitivity	Specificity
Dementia–control	71	0.97	0.84	0.87	0.97
MCI–control	84	0.85	0.54	0.92	0.63
Dementia–MCI	61	0.80	0.45	0.66	0.79

### Overlap in ACE-III scores across diagnoses

**Fig. 5:**
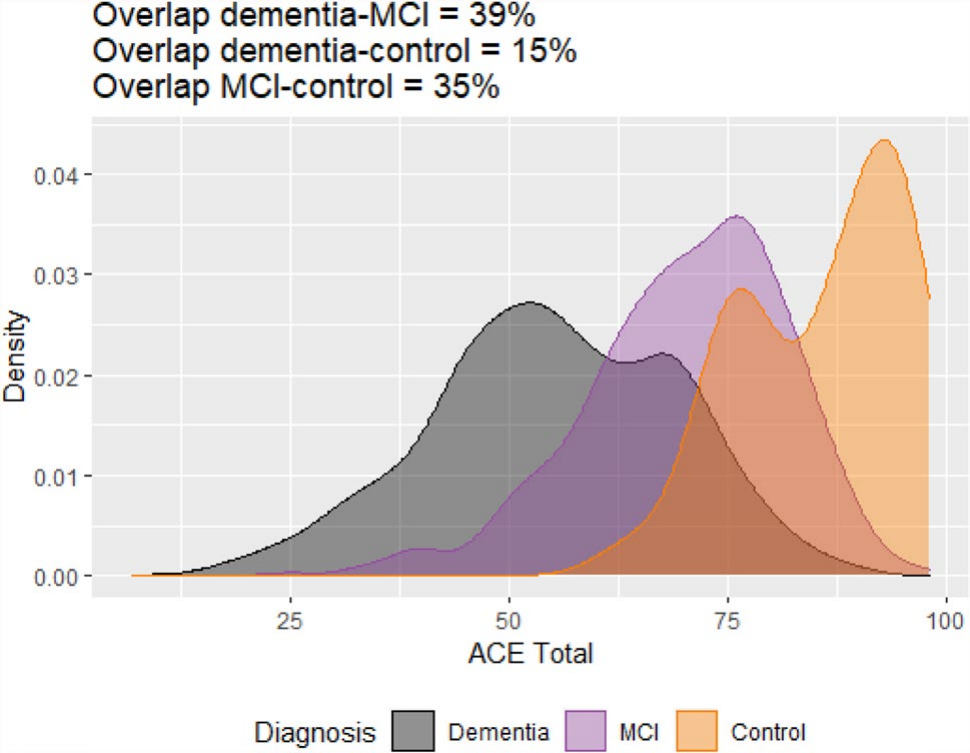
Kernel density estimations for ACE-III total between all dementias, MCI and controls. The overlap (%) represented by the shaded area is detailed above theplot

**Fig. 6:**
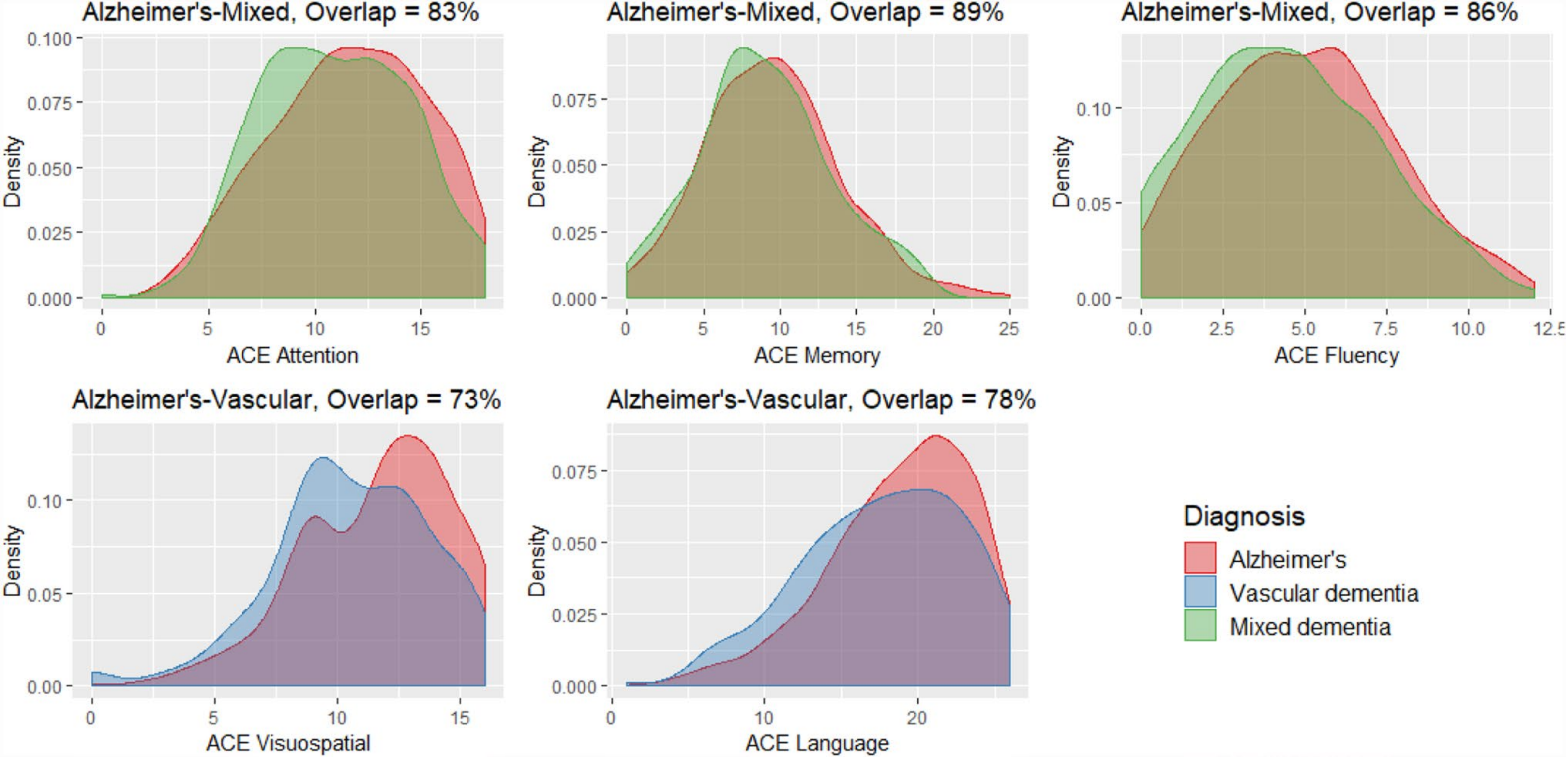
Kernel density estimations for ACE-III domains between dementia groups. The overlap (%) represented by the shaded area is detailed above each plot

The overlap in scores was very small (15%) when comparing total ACE-III between all dementias and the control group (Fig. 5). Roughly a third of total ACE-III scores overlapped between dementia–MCI (39%) and MCI–controls (35%) (Fig. 5). In contrast, the estimated overlap of densities between the dementia subtypes showed a high proportion of similarity (Fig. 6). Comparing densities for visuospatial and language, scores overlapped by 73% and 78%, respectively, for Alzheimer’s and Vascular dementia (Fig. 6). Similarly, the density plots for attention, memory and fluency between Alzheimer’s and mixed dementia overlapped to an even higher degree at 83%, 89% and 86%, respectively (Fig. 6).

### Pattern across ACE-III domain scores

**Fig. 7:**
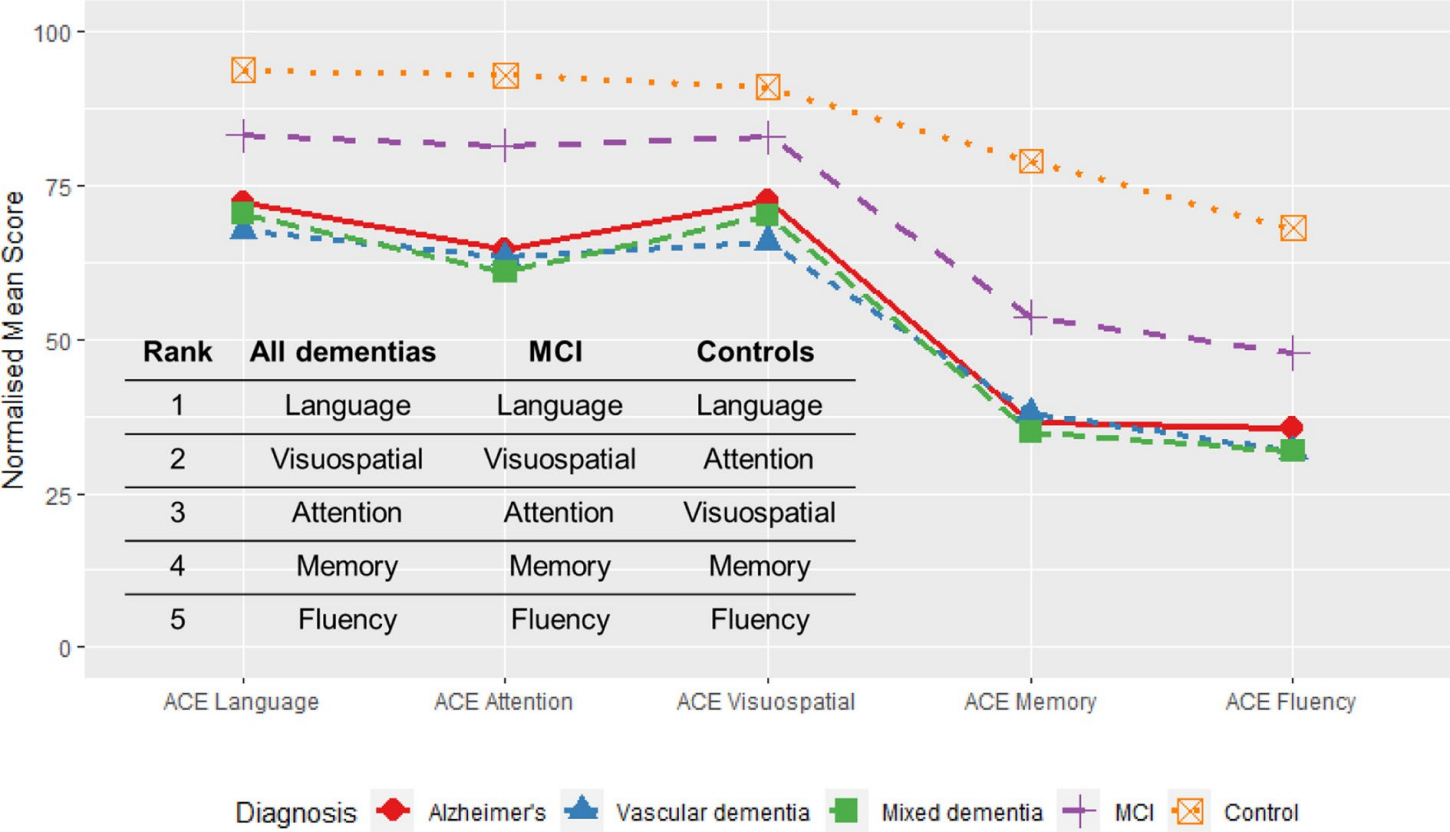
Normalised mean scores for each ACE-III domain across diagnostic groups, ordered from highest to lowest for the control group. Rank order from highest to lowest mean score shown for all dementias, MCI and controls (bottom left)

Across all dementias, MCI and controls, the pattern in normalised mean ACE scores is fairly consistent, with all groups scoring lowest in fluency and highest in language (Fig. 7). Mean scores were highest for controls, followed by MCI and the three dementia groups (Fig. 7). For the control group, the highest average score across domains was language, followed by attention, visuospatial, memory and fluency (Fig. 6). In contrast, the order was slightly different for all dementias and MCI in that the highest average score was language, followed by visuospatial, then attention memory and fluency (Fig. 6). On average, people with Alzheimer’s disease scored higher on attention, memory and fluency compared to those with mixed dementia (Fig. 7). The Alzheimer’s disease cohort scored better on average across visuospatial and language compared to the vascular dementia group (Fig. 7), and these differences in scores were statistically significant (*p* < 0.001 visuospatial, *p* < 0.05 language, Fig. 3) (Table 1).

**Fig. 8:**
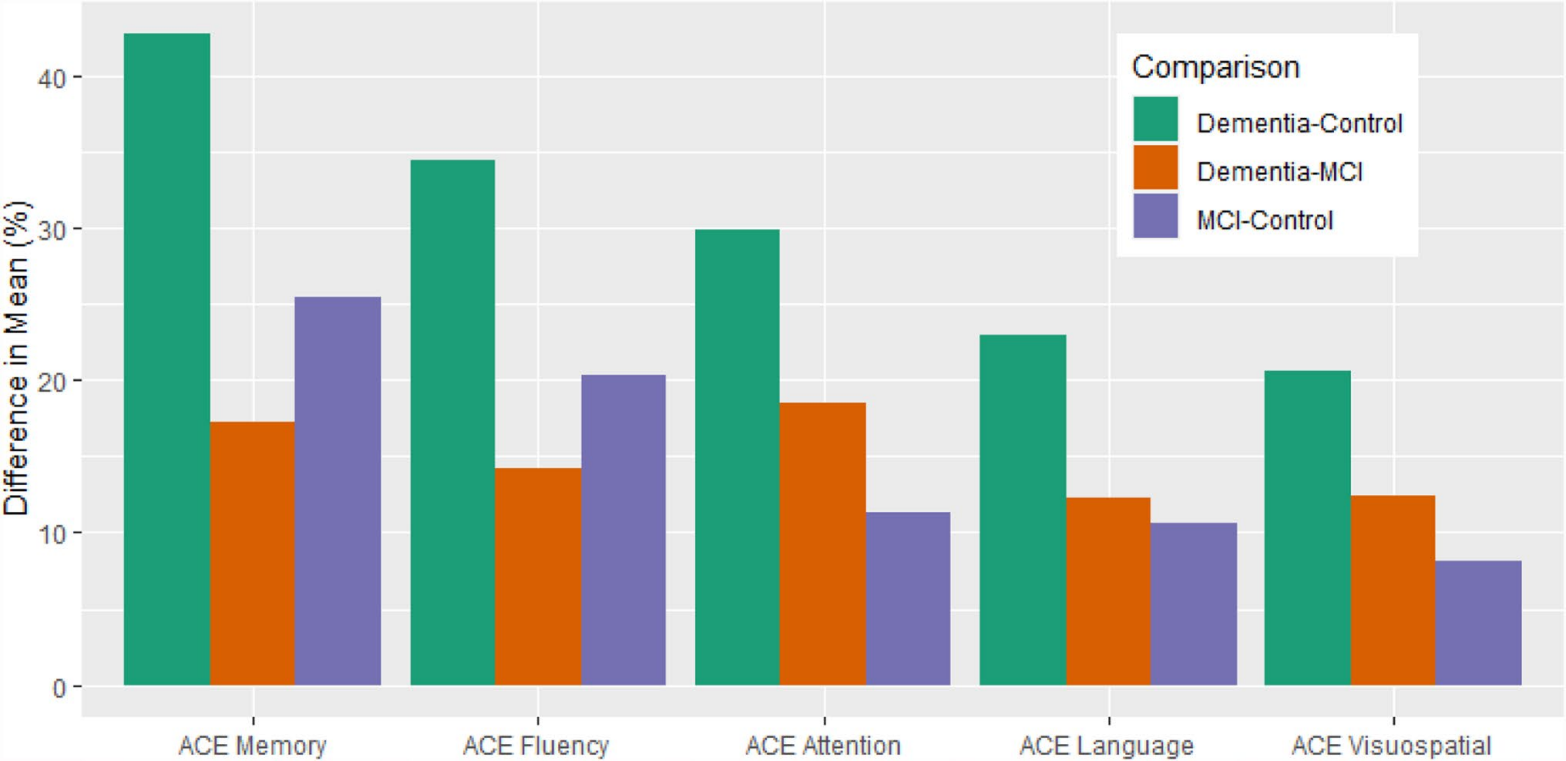
Difference in normalised mean scores across ACE-III domains for all dementias, MCI and controls. Ordered from highest to lowest for dementia–control comparison)

Comparing all dementias and the control group, the largest difference in average score was seen in the memory domain (> 40%) while the smallest difference was in visuospatial (~ 20%) (Fig. 8). The same order was evident when comparing MCI to the control group (Fig. 8) although the percentage differences were much smaller (~ 10–25%). In contrast, when comparing all dementias to MCI the largest difference in average score was in the attention domain and the smallest difference was in language; however, the percentage difference in average score was small, ranging from ~ 10 to 20% (Fig. 8).

## Discussion

This study utilised data from the NHSCT Memory Service. This unique resource is the first database of its kind and size and the first to examine the different patterns of performance across the ACE-III within such a diverse range of individuals with a dementia or MCI diagnosis compared with a control group.

In the NHSCT Memory Service, 62% of referrals were female. This figure is similar to the reported prevalence of dementia given 65% of people living with dementia in the UK are female (Prince et al. 2014). The gender ratio across different diagnostic categories varied; however, given 70% of people in the NHSCT Memory Service diagnosed with Alzheimer’s disease were female, which is relatively more than the proportional referral rate. Roughly 60% of mixed dementia and MCI diagnoses were female which highlights that there was a consistent proportion of males and females with MCI and mixed dementia. There was an almost even split of males and females diagnosed with vascular dementia, suggesting that males referred to the service are more likely to have vascular dementia than females referred to the service. This is consistent with the findings of the Rotterdam study, a large population-based study which found that regardless of age, vascular dementia was more prevalent in males compared to females (Ruitenberg et al. 2001).

The results of the present study demonstrate significant differences in ACE-III scores between people diagnosed with dementia, MCI and controls. People with dementia attending the NHSCT Memory Service perform significantly worse than the control group and people with MCI in terms of ACE-III total score and each of the domains. Roughly one third of total ACE-III scores overlapped between those with dementia compared to MCI and for the MCI and control groups. The overlap in total ACE-III scores between people with dementia and controls was even less (15%). These results suggest that the ACE-III is good at discriminating between dementia, MCI and people with no reported cognitive problems.

All three groups, including people with dementia, MCI and the control participants scored highest in language and lowest in memory and fluency on average. The pattern of normalised mean ACE-III domain scores highlighted an interesting pattern across groups. It was fairly consistent with the exception of attention which seemed to be disproportionately lower in both the dementia and MCI groups compared to controls. This suggests that relative impairment in attention is greater when there is a decline in cognition and this relative decline is greater in dementia than in MCI. This finding has the potential to be clinically helpful and needs further exploration.

Overall, intra-domain analysis revealed that within each of the groups of dementia, MCI and controls, there are similar abilities in the language and visuospatial domains, given there were no significant differences in scores between these domains. All groups also performed significantly lower on fluency than on language and visuospatial domains. Further analysis which looked at the intra-domain pairwise comparisons revealed some distinct patterns across these three groups. People with MCI and controls also have similar ability in the domain of attention in addition to language and visuospatial processing whereas, for people with dementia, ability in attention is significantly different from ability in the domains of language and visuospatial. This finding also has the potential to be clinically helpful and needs further exploration.

These results indicate different profiles of cognition, revealing distinct areas of cognitive decline progressing from a group reporting no cognitive problems (controls), through to those with MCI and people with dementia.

People with Alzheimer’s disease, vascular dementia and mixed dementia had similar distributions on ACE-III total and domain scores. There were statistically significant differences between those with Alzheimer’s and mixed dementia (attention, memory and fluency) and between individuals with Alzheimer’s and vascular dementia (visuospatial and language). While these differences were statistically significant, these results are not clinically significant or relevant, given that there was a high degree of overlap (> 73%) between these domain scores. Elamin and colleagues reported similar findings in their study, noting that between dementia subgroups there were few significant differences in ACE-III scores (Elamin et al. 2016).

The recommended total ACE-III cut-offs for differentiating early-onset dementia patients from healthy controls are 82 and 88 for research and screening, respectively (Hsieh et al. 2013). A recent review explored the diagnostic test accuracy of the ACE-III for dementia and found that the lower threshold of 82 provided better specificity with acceptable sensitivity (Beishon et al. 2019). However, the authors noted that the optimal cut-offs required future work and should be determined across a variety of settings such as secondary care services which would include the NHSCT Memory Service. Jubb and colleagues suggested a lower cut-off of 81 for better sensitivity and specificity in their sample of patients above 75 years of age presenting to a Memory Clinic in England (Jubb and Evans 2015). The authors also recommended taking other factors into consideration such as years of education when using ACE-III to aid diagnosis of dementia (Jubb and Evans 2015). In the present study, we identified a lower optimal cut-off of 71 for differentiating people with dementia from those with no cognitive impairment with acceptable sensitivity and high specificity. In practice, this means using a cut-off of 71 is highly likely to correctly predict that an individual doesn’t have dementia if dementia is not present. We also calculated an optimal cut-off score of 84 for distinguishing individuals with MCI from the control group with high sensitivity (0.92) but poor specificity (0.63). Given the control participants recruited for this study were individuals with no cognitive problems and otherwise would not normally be attending the NHSCT Memory Service, the cut-offs identified are more applicable for research purposes rather than screening.

As the ACE-III is not good at discriminating between different types of dementia, these results highlight the importance of the range of other factors that are taken into consideration when making a differential diagnosis of dementia. Other variables that have been shown to influence ACE-III scores include age and education level (Bruno and Vignaga 2019). Previous work has found that age significantly contributes to overall ACE-III score as those in older age groups perform worse across all domains of the ACE-III (Matias-Guiu et al. 2015; Cheung et al. 2015). Additionally, individuals with higher levels of education (> 11 years) perform better on the ACE-III compared to those with low levels of education (Matias-Guiu et al. 2015; Jubb and Evans 2015). Years of education are positively correlated with a person’s cognitive function as they age (Opdebeeck et al. 2016) and predict lower risk of dementia late in life (Lövdén et al. 2020). Some believe that increasing years of education builds cognitive reserve, which is a concept that the brain develops resilience that acts as a protective factor against loss through ageing and disease. Studies have linked this to better cognitive performance in people with dementia (Stern 2012), but also in other neurological diseases including Parkinson’s (Hindle et al. 2014) and multiple sclerosis (Santangelo et al. 2019).

The data confirm that the ACE-III total score alone cannot be used to diagnose dementia or distinguish between the different types of dementia. In addition, while there are some different patterns of performance across the domains of the ACE-III in the different types of dementia, it is not clear that a consistent pattern emerges to be helpful in making a decision about the specific diagnosis. This study was important because it confirms what clinicians already believed, that the ACE-III is an important tool to highlight that the person may have dementia or MCI but is not comprehensive enough to differentiate between the subtype of dementia. This is not surprising given that the ACE-III was not designed to differentiate between the range of dementias explored in this paper and instead to help differentiate between Alzheimer’s disease and frontotemporal dementia (Hsieh et al. 2013).

### Limitations

The ACE-III scores obtained on the test during the diagnostic process are the scores that were compared in the study to assess the reliability of ACE-III, and the test is only administered once. However, diagnosis is made on the basis of a comprehensive assessment as outlined in the Methods. This includes assessment based on clinical presentation, in combination with a medical report which usually comes from the GP that referred the patient to the service. The GP may have administered another cognitive screening tool such as MMSE, but this is not recorded in the NHSCT Memory Service database. Together, all of these factors are used by the psychiatrists to make a formal diagnosis. The analysis carried out did not control for age or education level, both of which have been shown to influence ACE-III scores, which is a significant limitation. The large sample sizes meant that some of the ACE-III domain scores across diagnostic groups were statistically significant; however, these results were not clinically significant. The sample size of the control group (*n* = 32) was considerably smaller than planned due to the coronavirus pandemic. Additionally, the control group were individuals recruited for the study with no cognitive impairment and normally would not be referred to the NHSCT Memory Service and thus may not be representative of people without dementia/ MCI referred to a memory service. Another limitation is the exclusion of data from certain groups. This study only looked at those who fully completed all sections of the ACE-III. There were many reasons people were unable to complete the ACE-III in full, such as tiredness, anxiety, distress, severity of cognitive difficulties, poor hearing or eyesight. A number of other groups were excluded including those with Lewy body dementia; frontotemporal dementia; unspecified diagnosis of dementia; other types of dementia and those with an uncertain diagnosis. These groups were taken out as the group sizes were not large enough for analyses or the exact diagnosis was not known at the time of assessment. Individuals who received no diagnosis of MCI or dementia were excluded as the majority of these people had other co-morbidities affecting cognitive function, and thus, they could not be included in the ‘control’ category. This study obtained data from the NHSCT Memory Service database in Northern Ireland; however, it is still only representative of data from a single location.

## Conclusion

This study analysed data from the NHSCT Memory Service database, a unique and comprehensive data repository detailing the outcome of dementia assessments. The aim of the study was to explore the reliability of the ACE-III in differentiating between dementia, MCI and controls, and whether the ACE-III is useful for making a differential diagnosis on the type of dementia. The results of this study suggest that the ACE-III is good for differentiating between dementia and MCI; however, the test is not reliable for discriminating between Alzheimer’s disease, vascular dementia and mixed dementia. Nonetheless, the ACE-III is a useful tool for clinicians that can help to make a dementia diagnosis in combination with other factors at assessment. Future work will involve utilising the NHSCT Memory Service database to analyse ACE-III scores for those groups that were excluded from the present study and determining the impact of factors that have been shown to influence ACE-III such as age, gender and years in education.
